# Perceived Disease Burden and Social Support Among Adults on Hemodialysis in Northern Colombia: A Cross-Sectional Study

**DOI:** 10.3390/nursrep16070217

**Published:** 2026-06-26

**Authors:** Yolima Judith Llorente Pérez, Jorge Luis Herrera Herrera, Edinson Oyola López, María Claudia Hernández López, Melisa Inés Peña Redondo

**Affiliations:** 1 Department of Nursing, University of Córdoba, Montería 230001, Colombia; jluisherrera@correo.unicordoba.edu.co (J.L.H.H.); melisapenar@correo.unicordoba.edu.co (M.I.P.R.); 2 Department of Nursing, University of Sinú, Montería 230001, Colombia; edinsonoyola@unisinu.edu.co (E.O.L.); mariachernandezl@unisinu.edu.co (M.C.H.L.)

**Keywords:** cost of illness, social support, chronic kidney disease, renal dialysis, cross-sectional studies, nursing

## Abstract

**Background/Objectives**: Chronic kidney disease and hemodialysis have a significant impact on patients’ lives. Social and clinical factors may influence perceived disease burden and the availability of social support, both of which are relevant for adherence and well-being. This study aimed to determine the relationship between perceived disease burden and social support among adults on hemodialysis in northern Colombia. **Methods**: A cross-sectional study was conducted on 183 patients receiving hemodialysis in northern Colombia. Disease burden was assessed using the GCPC-UN instrument and perceived social support using the Medical Outcomes Study Social Support Survey (MOS-SSS). Data were analyzed using descriptive statistics, nonparametric tests (Mann–Whitney U and Kruskal–Wallis), Spearman’s correlation, and a multivariate linear regression model with HC3 robust errors. **Results**: The mean age was 52.2 years (SD: 12.1), and 63.4% of participants were male. The overall disease burden score was 45.72 (SD: 16.47) out of a theoretical maximum of 144, with physical distress being the factor that contributed most to it (mean: 20.03; SD: 6.83). Functional social support was moderate (median: 72; IQR: 63–80). A significant inverse correlation was found between social support and disease burden (Spearman’s rho = −0.160; *p* = 0.03). In the multivariate model, time on hemodialysis was associated with a higher burden (a 0.628-point increase per 12 months; 95% CI: 0.022–1.234; *p* = 0.04), while age showed a non-significant inverse trend. **Conclusions**: When assessing the burden of disease, patients on hemodialysis primarily experience physical distress and perceive a moderate level of social support. A longer duration of dialysis is associated with an increase in the perceived burden, while social support showed a weak inverse correlation with the burden of disease in the unadjusted analysis; however, this association was not confirmed as statistically significant in the multivariable model.

## 1. Introduction

Chronic kidney disease (CKD) is a major global public health problem, affecting approximately 10% of the population and ranking among the leading causes of global mortality [1]. Globally, it is estimated that more than 2.5 million people are receiving dialysis treatment, a figure that could double by 2030 if current epidemiological trends continue [2].

In Latin America, CKD has shown steady growth, primarily due to an aging population and an increase in metabolic risk factors [3]. In Colombia, recent reports indicate a similar trend; patients who progress to hemodialysis have a multimorbidity profile characterized primarily by hypertension (AH) and type 2 diabetes mellitus (DM2) [4].

In northern Colombia, it has been reported that 48.6% of dialysis patients have AH, while 32.8% have DM2, AH, and CKD simultaneously [4]. This coexistence of conditions increases the complexity of clinical management [5] and is associated with greater physical discomfort [6] and limitations in patients’ biopsychosocial adjustment [7].

From a clinical and functional perspective, the progression of CKD leads to irreversible deterioration of kidney function, ultimately requiring renal replacement therapy, with hemodialysis being the most commonly used modality [8]. However, although this treatment is essential for survival, it imposes a demanding treatment regimen that significantly affects patients’ daily lives [9].

This process is related to the concept of disease burden [10], understood as a multidimensional construct that includes the physical, psychological, and socioeconomic impact perceived by the individual [10]. In this context, studies such as that by Jhamb et al. have shown that the symptomatic burden in hemodialysis patients can be comparable with that observed in advanced cancer, with an average of up to nine persistent symptoms, among which fatigue and chronic pain are highly prevalent [6,11]. Consequently, the perceived burden transcends clinical symptoms and is a subjective phenomenon that affects the individual’s emotional stability and social functioning.

In this context, the literature has explored the role of social determinants in coping with illness. Several studies have shown that low levels of social support are associated with a higher risk of mortality and a greater emotional burden [12].

In resource-limited settings, a lack of practical and emotional support increases patients’ vulnerability and makes it difficult for them to adhere to dietary restrictions and dialysis regimens [13]. In contrast, a higher level of social support has been identified as a significant external resource and a protective factor against the stressors associated with chronic illness [14]. This is defined as the network of relationships that provides emotional, informational, and instrumental support [14]. However, in contexts with marked socioeconomic inequalities the effectiveness of this support may be limited by structural constraints, which could increase the perceived burden of the disease [15,16,17].

Despite the importance of these constructs, a knowledge gap persists regarding the interaction between the perceived burden of illness and social support among hemodialysis patients, particularly in specific Latin American contexts. Although international cohort studies have examined how social support correlates with quality of life and clinical outcomes [18,19,20], these findings cannot be directly extrapolated to the Colombian Caribbean. This region is characterized by unique structural inequalities, where 77% of patients are enrolled in the subsidized health system and 66.7% reside in the lowest socioeconomic strata [15,16,17]. Therefore, it has not been established whether functional social support significantly mitigates the multidimensional impact of the burden of disease in patients with prolonged exposure to treatment under these specific socioeconomic and cultural constraints. Examining these variables in this specific population is warranted to guide tailored local public health policies and nursing interventions.

Therefore, the aim of this study was to determine the relationship between perceived disease burden and social support among adults on hemodialysis in northern Colombia. To address this objective, the primary hypothesis was that greater perceived social support would be associated with a lower disease burden among adults on hemodialysis. Additionally, the study explored how this burden varied according to sociodemographic and clinical variables.

## 2. Materials and Methods

### 2.1. Design, Display, and Sampling

A quantitative cross-sectional study with a correlational design was conducted. Based on previous evidence, the primary hypothesis was that greater perceived social support would be associated with a lower disease burden among adults on hemodialysis. Secondarily, the study explored how this burden varied according to sociodemographic and clinical variables.

This study followed the recommendations of the STROBE (Strengthening the Reporting of Observational Studies in Epidemiology) guidelines for observational studies to ensure quality and transparency in epidemiological research [21]. This design allowed for an analysis of the relationship between sociodemographic and clinical variables and the burden of disease and perceived social support.

Simple random sampling was used to ensure the representativeness of the population. The source population consisted of 350 patients actively enrolled in the hemodialysis program during the study period. Using the institutional registry, each patient was assigned a sequential number, and selection was performed using a random number generator until the sample size was reached. When a selected patient did not meet the eligibility criteria or declined to participate, a new random selection was made from the same list.

The sample size was estimated based on an accessible population of 350 patients, with a 95% confidence level and a 5% margin of error, resulting in 183 participants. Since the primary analytical objective included correlation and regression, this calculation should be interpreted as an estimate based on population precision and not as a specific power calculation to detect a predetermined correlation coefficient. It is acknowledged that the sample calculation was based on a finite population and not on a formal hypothesis of correlation or regression.

Recruitment took place at a healthcare facility that treats patients with chronic kidney disease undergoing renal replacement therapy, located in Monteria, Colombia, between January and June 2024. Patients were approached before or after their scheduled hemodialysis session. Subjects were randomly selected based on eligibility criteria until the defined sample size was reached.

Of the 250 patients evaluated, 200 met the inclusion criteria; of these, 17 were excluded due to lack of time, and the final sample analyzed consisted of 183 participants.

Individuals over the age of 18 who were actively receiving renal replacement therapy were included. Patients who were hospitalized at the time of recruitment or those diagnosed with cognitive or neurological disorders that could limit their participation in this study were excluded. This information was verified by reviewing the medical record and confirming it with the family caregiver. Patients with literacy barriers or an inability to provide informed consent were also excluded.

### 2.2. Data Collection Procedures

To collect the data, each participant was informed of the study’s objective, and their informed consent was subsequently obtained. Members of the research team administered the assessment tools to each participant who met the eligibility criteria.

A demographic form designed by the research team was used to collect sociodemographic and clinical variables, which included age, sex, educational level, religion, socioeconomic status, health insurance affiliation, place of origin, socioeconomic stratum, underlying medical condition, duration on hemodialysis, and presence of medical complications.

The disease burden was assessed using the Chronic Disease Burden for Patients (GCPC-UN) instrument, which has been validated in the Colombian context and has a Cronbach’s alpha of 0.925 [22]. The instrument consists of 48 items (scored between 0 and 144 points) distributed across three dimensions: emotional and spiritual suffering (15 items: scored on a scale of 0 to 45 points), physical discomfort (18 items: scored between 0 and 54 points), and socio-family and cultural disturbances (15 items: scored on a scale of 0 to 45 points). A 4-point Likert scale was used with the following response options: never (0), rarely (1), often (2), and always (3). Each item assesses the frequency, intensity, and perceived threat associated with the condition. Higher scores indicate a greater perceived burden of disease.

Social support was measured using the Medical Outcomes Study Social Support Survey (MOS-SSS), validated in the Colombian population [23,24], with a Cronbach’s alpha of 0.941. The instrument consists of 20 items. The first assesses the size of the social network, and the remaining 19 are grouped into four dimensions: emotional support (items 3, 4, 8, 9, 13, 16, 17, and 19), instrumental support (items 2, 5, 12, and 15), positive social interaction (items 7, 11, 14, and 18), and affective support (items 6, 10, and 20). Each item is rated on a 5-point Likert scale: never, rarely, sometimes, most of the time, and always. Scores were standardized on a scale from 0 to 100 to facilitate comparison across dimensions; higher values indicated a higher level of perceived social support. For the qualitative classification of the MOS-SSS, the cut-off scores reported for people with chronic disease in the Colombian population were used: overall low 19–62, moderate 63–81, and high 82–95; the dimensional cut-offs were applied according to the original proposal/validation used [23,24].

### 2.3. Statistical Analysis

Statistical analysis was performed using IBM SPSS Statistics, version 27 (IBM Corp., Armonk, NY, USA). Categorical variables were described using absolute frequencies and percentages. Continuous variables were summarized as means and standard deviations (SDs) or as medians and interquartile ranges (IQRs), depending on their distribution. Time on hemodialysis was standardized in months for analytical purposes.

The total scores of the MOS-SSS and the frequency dimension of the GCPC-UN are presented as medians (IQRs) due to the non-normal distribution of the data. A bivariate analysis was performed between sociodemographic and clinical variables and the instrument scores. The Mann–Whitney U test was used for comparisons between two groups, and the Kruskal–Wallis test for three or more groups. A *p*-value of <0.05 was considered statistically significant.

The presence of missing data was assessed for each variable prior to analysis. Observations with incomplete data were handled using complete-case analysis in the multivariate models.

A linear regression model with HC3-type robust standard errors was constructed, in which the dependent variable was the total frequency score of the GCPC-UN. The reference categories were female gender, urban origin, marital status with a partner (married or cohabiting), middle socioeconomic status (2–3), an underlying condition of hypertension associated with chronic kidney disease (AH + CKD), and absence of complications related to hemodialysis. The “underlying condition” variable was recoded into three categories: (1) hypertension (HTN) + chronic kidney disease (CKD), as the reference group; (2) conditions with type 2 diabetes (T2D), which included T2D + HTN + CKD, T2D + CKD, and HTN + T2D + CKD + heart disease; and (3) other comorbidities, which included HTN + CKD + thyroid disease, HTN + CKD + lupus, and HTN + CKD + arthritis. Marital status was recoded as “in a relationship” (married/cohabiting) and “not in a relationship” (single/separated/widowed). Complications were recoded as “none” versus “some complications.”

The total MOS-SSS score was included in the model in 10-point increments, age in 10-year increments, and duration of hemodialysis in 12-month increments.

Adjustment covariates were selected based on their clinical and epidemiological relevance as potential confounding factors, including age, sex, place of origin, marital status, socioeconomic status, duration of hemodialysis, underlying medical conditions, and complications related to hemodialysis.

### 2.4. Ethical Considerations

This study was approved by the Research Committee of the Faculty of Health Sciences at the University of Córdoba (Minutes No. 05 of 26 September 2023). In accordance with Resolution No. 8430 of 1993 issued by the Colombian Ministry of Health [25], this research was classified as low risk. This study also followed the ethical principles of the Declaration of Helsinki.

Before participation, all individuals received information about this study’s objectives, procedures, risks, and benefits, and provided written informed consent. The consent process included a verbal explanation in clear language to ensure participants’ understanding.

Participants were assigned alphanumeric codes to ensure confidentiality and anonymity. Data were used exclusively for research purposes, stored in secure databases, and accessed only by the research team.

## 3. Results

### 3.1. Participant Characteristics

The mean age of the participants was 52.2 years (SD: 12.1), with a median of 54 years (IQR: 46–61). The sample was predominantly male (63.4%). Most participants were from urban areas (67.8%), had a primary school education (55.2%), were Catholic (71.6%), and lived in a common-law relationship (41.0%).

Regarding socioeconomic status, the largest proportion belonged to the subsidized health insurance scheme (77.0%) and socioeconomic stratum 1 (66.7%), followed by stratum 2 (27.3%). Regarding occupation, the group not engaged in work predominated (59.0%), followed by those who were employed (26.2%) and housewives (13.1%).

From a clinical perspective, the most common combination of underlying conditions was hypertension and chronic kidney disease (48.6%), followed by the coexistence of type 2 diabetes mellitus, hypertension, and chronic kidney disease (32.8%), and type 2 diabetes mellitus with chronic kidney disease (11.5%). Other combinations of comorbidities were less common.

The duration of hemodialysis was 59.8 months on average (SD: 58.9) and 40 months at the median (IQR: 14–74), indicating wide variability in the treatment duration. Regarding complications associated with hemodialysis, 54.6% of participants reported no adverse events; among those who did experience complications, infection was the most common (31.1%), followed by thrombosis (7.7%) and bleeding (6.6%) (Table 1).

It should provide a concise and precise description of the experimental results, their interpretation, and the experimental conclusions that can be drawn.

### 3.2. Disease Burden and Perceived Social Support

Regarding the burden of chronic illness, the dimension of physical discomfort had the highest means for frequency (20.03; SD: 6.83), intensity (3.02; SD: 2.02), and threat (2.44; SD: 2.02). In contrast, the dimension of socio-family and cultural alterations showed lower values for frequency (12.29; SD: 6.07), intensity (2.02; SD: 2.10), and threat (1.48; SD: 1.60).

Overall, the disease burden had a mean frequency of 45.72 out of a theoretical maximum of 144 (SD: 16.47), a mean intensity of 7.23 (SD: 4.54), and a mean severity of 5.80 (SD: 4.10) (Table 2).

In the assessment of perceived social support, the structural component of the MOS showed a mean of 2.3 (SD: 2.1) friends and 3.6 (SD: 2.0) family members, with medians of 2 (IQR: 1–3) and 3 (IQR: 2–4), respectively. In the functional component, the overall MOS score was 72.4 (SD: 13.8), with a median of 72 (IQR: 63–80).

When analyzing the MOS dimensions, the highest mean score corresponded to emotional/informational support (30.3; SD: 6.0), followed by positive social interaction (15.5; SD: 3.1), instrumental support (15.3; SD: 3.4), and affective support (11.3; SD: 2.8). The medians ranged from 12 for affective support (IQR: 9–14) to 30 for emotional/informational support (IQR: 26.5–34) (Table 3).

Regarding the overall social support classification, the moderate level was the most common (54.1%), while the low and high levels each accounted for 23.0%. In the emotional/informational support dimension, 60.7% of participants fell into the moderate level.

For instrumental support, 49.2% were classified at the moderate level, 27.9% at the high level, and 23.0% at the low level. In the dimension of positive social interaction, 47.5% fell into the medium level, 38.8% into the high level, and 13.7% into the low level. Similarly, in affective support, 34.4% were classified at the medium level, 38.8% at the high level, and 26.8% at the low level.

### 3.3. Association Between Sample Characteristics, Disease Burden, and Social Support

In the bivariate analysis, the total GCPC-UN frequency score differed according to marital status (*p* = 0.016), underlying medical condition (*p* = 0.049), and the presence of complications related to hemodialysis (*p* < 0.001). The median burden was higher among single participants (51.0; IQR: 43.5–57.0) compared with those who were married or in a domestic partnership. It was also higher in those who reported hemodialysis-associated infections (49.0; IQR: 45.0–55.0) compared with those who had no complications (43.0; IQR: 33.8–51.2).

A trend toward differences by socioeconomic status was observed (*p* = 0.088), while no statistically significant differences were identified by sex, place of origin, educational level, religion, health insurance status, or occupation (Table 4).

Although no statistically significant differences were observed by religion, participants who were Jehovah’s Witnesses had a descriptively higher median burden than the Catholic and Evangelical groups; however, this finding should be interpreted with caution due to the small size of this subgroup.

Regarding perceived social support, the overall MOS-SSS score differed according to place of origin (*p* < 0.001), marital status (*p* < 0.001), insurance status (*p* = 0.028), and the presence of complications related to hemodialysis (*p* = 0.001). Participants from urban areas had a higher median social support score than those from rural areas (74.5; IQR: 66.8–85.2 vs. 67.0; IQR: 60.0–75.0). Likewise, lower medians were observed among single and separated participants, as well as among those enrolled in the special health insurance plan.

Trends toward differences were also identified according to sex (*p* = 0.058), occupation (*p* = 0.064), and underlying medical condition (*p* = 0.091) (Table 4).

In the Spearman correlation analysis, age showed a weak negative correlation with the total frequency score on the GCPC-UN (rho = −0.148; *p* = 0.046). In contrast, duration of hemodialysis was not correlated with the frequency-based disease burden (rho = 0.030; *p* = 0.686) (Table 5).

Regarding the overall MOS-SSS score, age showed no correlation (rho = −0.005; *p* = 0.946). Similarly, duration of hemodialysis was not correlated with perceived social support (rho = 0.087; *p* = 0.240).

**Figure 1:**
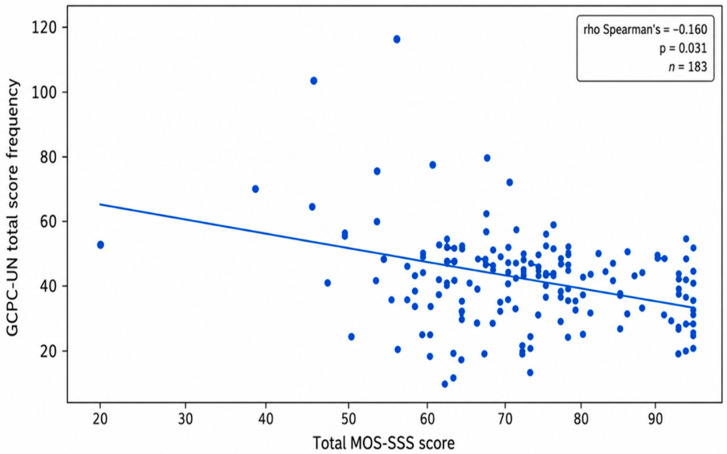
Relationship between the total MOS score and the total frequency of GCPC-UN in the study sample (*n* = 183). Note: Relationship between the total MOS-SSS score (X-axis; higher values indicate greater perceived social support) and the total GCPC-UN frequency score (Y-axis; higher values indicate a greater disease burden). Each point represents a participant, and the solid line indicates the linear trend.

Figure 1 shows a weak inverse relationship between perceived social support (total MOS-SSS score) and the frequency of disease burden (total GCPC-UN score). Higher scores for social support are associated with lower scores for disease burden. This relationship is reflected in the downward slope of the fitted line and in the Spearman correlation coefficient (rho = −0.160; *p* = 0.031) among the 183 participants evaluated.

In the adjusted multivariate model, the duration of hemodialysis was the only variable associated with the GCPC-UN frequency score (*p* = 0.042). For every additional 12 months of treatment, the score increased by an average of 0.628 points (β = 0.628; 95% CI: 0.022 to 1.234). Although the duration of hemodialysis did not show a significant correlation in the bivariate analysis, it was associated in the adjusted model. This discrepancy suggests a possible confounding effect or statistical suppression by other covariates; therefore, this finding should be interpreted with caution.

Overall social support, measured by the total MOS-SSS score, showed an inverse association with disease burden; however, this was not statistically significant (*p* = 0.101). For every 10-point increase in the MOS-SSS, the GCPC-UN frequency score decreased by an average of 3.216 points (β= −3.216; 95% CI: −7.054 to 0.622).

Similarly, age showed a non-significant inverse association (*p* = 0.067); for every 10-year increase, the GCPC-UN score decreased by an average of 2.09 points (β= −2.09; 95% CI: −4.322 to 0.142).

No statistically significant associations were observed between the frequency of disease burden and the variables of sex, place of origin, marital status, socioeconomic status, underlying condition, or the presence of complications arising from hemodialysis. In the case of the low socioeconomic status group, a positive coefficient was observed (β = 3.938; 95% CI: −1.014 to 8.891; *p* = 0.119) (Table 6).

Although marital status and the presence of complications showed an association with the overall MOS-SSS score in the bivariate analysis, these variables lost statistical significance in the adjusted model. This pattern could be due to confounding factors related to other included covariates, partial multicollinearity with treatment duration, or limited precision due to sample size; therefore, these results should be interpreted with caution (Table 6).

## 4. Discussion

### 4.1. Key Findings

The analysis of this study revealed an inverse correlation between perceived social support and the burden of illness, albeit of weak magnitude. This finding suggests that higher levels of social support are associated with a lower perceived burden among hemodialysis patients. Consistent with these results, as described in the literature [26], it is proposed that social support may act as a protective factor against stressors associated with chronic illness. In the context of northern Colombia, although functional social support was at an intermediate level, the results indicate that it could play a significant role in adaptation to dialysis treatment.

However, it is essential to note that, although this inverse relationship was statistically significant in the unadjusted bivariate analysis (rho = −0.160, *p* = 0.031), it did not retain statistical significance when analyzed independently within the fully adjusted multivariate model (β = −3.216, *p* = 0.101). This discrepancy suggests that the potential mitigating capacity of social support does not operate in isolation; rather, its effect is likely shared or conditioned by other dominant clinical and sociodemographic factors, such as treatment duration. Therefore, although social support remains a valuable contextual resource for adaptation, its role as an absolute independent predictor of disease burden should be interpreted with due scientific caution.

### 4.2. Analysis of the Perceived Disease Burden

When assessing the burden of disease as a single concept, the results of this study indicate a greater impact on physical health, consistent with findings reported in other Latin American contexts [3]. In these settings, chronic kidney disease is associated not only with higher mortality but also with a significant impact on years of healthy life lost.

In the Colombian context, the findings are consistent with previous studies that identify physical discomfort as the most affected dimension in people with chronic diseases [17]. In this study, this dimension had the highest scores in frequency, intensity, and threat, reinforcing its relevance to the experience of the disease.

In contrast with reports from cities such as Bogotá [26] and Medellín [27], participants from northern Colombia reported lower levels of burden in the socio-family dimension [27]. This finding may be related to family and social dynamics specific to the regional context; however, this interpretation should be considered with caution, as the study did not directly assess cultural or family structure variables.

### 4.3. Perceived Social Support Behavior

With regard to perceived social support, the intermediate level identified in this sample is consistent with national macroregional data from Colombia that used the MOS-SSS questionnaire in populations with chronic diseases [26]. Specifically, the literature shows that, while chronic patients in Colombia perceive adequate baseline support, cohorts in the Caribbean region tend to report lower average scores compared to other geographic areas, such as the Amazon and Orinoquía regions [26]. This contextualizes the current findings within an established regional baseline, highlighting that, although informal support networks exist, their perceived effectiveness in northern Colombia remains moderate, reflecting the documented regional challenges in fully meeting the socio-family needs of people living with chronic conditions. In this regard, when analyzing the internal components of such support, the literature suggests that, in the Colombian context, the emotional and affective dimensions tend to score higher than instrumental support [28].

This pattern was also observed in the present study, where the dimensions related to emotional support and social interaction showed higher values than those for instrumental support.

Although a significant proportion of the participants belonged to low socioeconomic strata, affective support remained relatively high. This finding suggests that even in contexts of material deprivation, forms of non-instrumental support may persist within close social networks.

When comparing these results with studies conducted in other Latin American contexts, where higher levels of social isolation have been reported among hemodialysis patients [29,30], the population analyzed in this study showed relatively higher levels of social support. These differences could be related to contextual factors or characteristics of support networks, although they were not directly assessed in this study.

### 4.4. The Influence of Sociodemographic and Clinical Factors

The sociodemographic profile helps to contextualize this study’s findings. The predominance of participants from lower socioeconomic strata and the subsidized health plan reflects a population facing structural vulnerability. However, in the multivariate model, socioeconomic status was not significantly associated with the burden of disease, suggesting that, in this sample, the perceived burden may be more closely related to clinical factors than to economic status.

A notable statistical finding in this study is the discrepancy between the non-significant bivariate correlation (rho = 0.030, *p* = 0.686) and the significant multivariate regression coefficient (β = 0.628, *p* = 0.042) for the duration of hemodialysis. This pattern could suggest a potential suppression effect or statistical confounding. In the bivariate analysis, the unadjusted relationship between treatment duration and perceived disease burden may have been influenced by concurrent sociodemographic and clinical variables. However, once these potential confounding factors were simultaneously controlled for within the multivariate model, a significant independent association emerged, suggesting that greater exposure to hemodialysis could contribute to an increase in the perceived burden of disease when other covariates are held constant.

In this regard, time on hemodialysis was the only variable associated with the burden of disease in the adjusted model. For every additional 12 months of treatment, the burden score increased by an average of 0.628 points. This finding suggests that prolonged exposure to treatment may be related to a progressive increase in the perceived burden.

These results are consistent with those reported in previous studies describing an increase in burden associated with the chronicity of treatment and its cumulative effects [31,32]. However, they partially differ from what has been observed in some European registries, where a stabilization of the perceived burden has been described after the first few years of treatment [33]. These differences could be related to variations in health systems, access to support resources, or the characteristics of the populations studied.

In the context of northern Colombia, the findings suggest that the duration of treatment may be a significant factor in the experience of the disease. However, this interpretation should be viewed with caution, given this study’s cross-sectional design, which does not allow for the establishment of causal relationships or the assessment of changes over time.

Meanwhile, age showed a non-significant inverse association with the disease burden, consistent with studies suggesting that older patients tend to report a lower emotional burden [34]. This pattern could be related to differences in coping mechanisms, although this was not directly assessed in this study.

### 4.5. Implications for Public Health and Clinical Practice

From a public health perspective, these findings provide relevant insights into the development of comprehensive care strategies for patients on hemodialysis. The association between treatment duration and higher levels of burden suggests that support interventions could be tailored according to the therapy duration, with a progressive emphasis on patients who have been on dialysis for a longer period.

In the clinical setting, the results support the incorporation of interdisciplinary interventions, particularly from nursing and mental health, aimed at managing perceived burden and strengthening social support. Likewise, promoting the involvement of the family network could help improve the patient experience and reduce the risk of isolation.

Finally, the timely identification of physical discomfort, as the primary dimension of burden, could facilitate early interventions aimed at improving well-being and treatment adherence. However, these implications should be interpreted while considering this study’s cross-sectional design.

### 4.6. Limitations and Future Research Directions

Despite the significance of these findings, several limitations must be acknowledged. First, the cross-sectional design prevents the establishment of causal relationships or the assessment of temporal changes among the variables. Second, although probabilistic sampling was used, data collection was restricted to a single healthcare center, which limits the generalizability of the results and precludes direct replication.

Furthermore, the sample size was initially calculated for estimating proportions rather than for correlation analysis; therefore, the statistical power for Spearman’s tests may be limited, and the discrepancy observed between the unadjusted bivariate analysis and the multivariate model regarding treatment duration should be interpreted as a methodological limitation.

Regarding data collection instruments, all questionnaires were administered in Spanish via self-report. Although no explicit literacy barriers or requests for assistance were recorded during administration, the high concentration of participants in socioeconomic stratum 1 and with a primary school education level represents a potential contextual bias.

In addition, the GCPC-UN lacks universally validated cutoff points for classifying scores as low, moderate, or high; therefore, describing the burden in this sample as “moderate” reflects an operational interpretation rather than a validated clinical threshold. Finally, cultural, family, and coping variables were not controlled for. Future studies should employ longitudinal and multicenter designs with adequate statistical power for multivariate models, along with qualitative approaches to capture patients’ subjective experiences.

## 5. Conclusions

Adults on hemodialysis in northern Colombia experience mainly physical discomfort in terms of their disease burden. These findings partially support the study’s hypotheses; while treatment duration was significantly and positively associated with higher levels of burden in the multivariate analysis, functional social support showed a significant inverse relationship only in the unadjusted bivariate framework, losing its independent significance when adjusted for other covariates. Therefore, while social support highlights a potential mitigating role for this particular study group, it should be considered a contextual factor rather than an independent predictor of burden.

These findings highlight the importance of considering clinical and social factors in the care of hemodialysis patients and suggest the need for comprehensive care strategies such as early management of physical distress, ongoing psychological support, and active strengthening of family support networks aimed at improving patient well-being and experience.

## Figures and Tables

**Table 1:** Sociodemographic and clinical characteristics of the study population (*n* = 183).

Variable	Value ^1^
**Age (years)**	52.2 ± 12.1; 54 [46–61]
**Sex**	
Female	67 (36.6)
Male	116 (63.4)
**Origin**	
Urban	124 (67.8)
Rural	59 (32.2)
**Education**	
Elementary	101 (55.2)
High school	54 (29.5)
Technical/vocational	13 (7.1)
Vocational	14 (7.7)
Graduate	1 (0.5)
**Religion**	
Catholic	131 (71.6)
Evangelical	36 (19.7)
Jehovah’s witness	16 (8.7)
**Marital status**	
Single	23 (12.6)
Married	67 (36.6)
Cohabiting	75 (41.0)
Separated	9 (4.9)
Widowed	9 (4.9)
**Healthcare system**	
Subsidized	141 (77.0)
Contributory	23 (12.6)
Special	19 (10.4)
**Socioeconomic stratum**	
Stratum 1	122 (66.7)
Stratum 2	50 (27.3)
Stratum 3	11 (6.0)
**Occupation**	
Housewife	24 (13.1)
Student	3 (1.6)
Employed	48 (26.2)
Unemployed	108 (59.0)
**Underlying conditions**	
AH ^2^ + CKD ^3^	89 (48.6)
DM2 ^4^ + AH + CKD	60 (32.8)
DM2 + CKD	21 (11.5)
AH + DM2 + CKD + heart disease	3 (1.6)
AH + CKD + thyroid disease	3 (1.6)
AH + CKD + lupus	5 (2.7)
AH + CKD + arthritis	2 (1.1)
**Duration of hemodialysis (months)**	59.8 ± 58.9; 40 [14–74]
**Complications related to the hemodialysis process**	
None	100 (54.6)
Infection	57 (31.1)
Blood clots	14 (7.7)
Bleeding	12 (6.6)

^1^ Values are presented as *n* (%) for categorical variables and as means ± standard deviation; medians [interquartile range] for continuous variables. ^2^ AH: hypertension. ^3^ CKD: chronic kidney disease. ^4^ DM2: type 2 diabetes mellitus.

**Table 2:** Burden of chronic disease by dimension (*n* = 183).

Dimensions	Frequency (Mean ± SD)	Intensity (Mean ± SD)	Threat (Mean ± SD)
Emotional and spiritual distress	13.40 ± 6.22	2.19 ± 1.94	1.89 ± 1.79
Physical discomfort	20.03 ± 6.83	3.02 ± 2.02	2.44 ± 2.02
Social, family, and cultural disruptions	12.29 ± 6.07	2.02 ± 2,10	1.48 ± 1.60
Overall burden of disease	45.70 ± 16.50	7.20 ± 4.50	5.80 ± 4.10

Note: The values are presented as means ± standard deviation.

**Table 3:** Perceived social support (*n* = 183).

Variable	*n* (%)	Mean ± SD	Median (IQR)	Min.–Max.
**Structural social support (network)**				
Number of friends		2.30 ± 2.10	2 [1–3]	0–10
Number of family members		3.60 ± 2.00	3 [2–4]	0–10
**Functional social support**				
MOS overall score		72.40 ± 13.80	72 [63–80]	21–95
**Dimensions of social support**				
Emotional/informational support		30.30 ± 6.00	30 [26.5–34]	12–40
Instrumental support		15.30 ± 3.40	15 [13–18]	4–20
Positive social interaction		15.50 ± 3.10	16 [13.5–18]	6–20
Affective support		11.30 ± 2.80	12 [9–14]	3–15
**MOS-SSS overall score–qualitative**				
Low	42 (23.0)			
Medium	99 (54.0)			
High	42 (23.0)			
**Emotional/informational support**				
Low	25 (13.7)			
Medium	111 (60.7)			
High	47 (25.7)			
**Instrumental support**				
Low	42 (23.0)			
Medium	90 (49.1)			
High	51 (27.9)			
**Positive social interaction**				
Low	25 (13.7)			
Medium	87 (47.5)			
High	71 (38.8)			
**Emotional support**				
Low	49 (26.8)			
Medium	63 (34.4)			
High	71 (38.8)			

Note: The values are presented as *n* (%), means ± standard deviation, medians [interquartile range], and minimum–maximum values. For the qualitative classification of the MOS-SSS, the following cut-off points were used: low 19–62, moderate 63–81, and high 82–95.

**Table 4:** Bivariate analysis of the relationship between sociodemographic and clinical variables and the total scores on the instruments (*n* = 183).

Variable/Category	*n*	MOS-SSS Total, Median (IQR)	*p*-Value	GCPC-UN Total Frequency, Median (IQR)	*p*-Value
**Sex**			0.058		0.776
Female	67	70.0 [62.0–78.5]		47.0 [37.5–53.0]	
Male	116	74.0 [64.8–80.0]		47.0 [36.0–52.0]	
**Origin**			<0.001		0.473
Urban	124	74.5 [66.8–85.2]		48.0 [36.8–52.0]	
Rural	59	67.0 [60.0–75.0]		43.0 [35.5–54.0]	
**Education level**			0.647		0.943
Elementary school	101	72.0 [63.0–79.0]		45.0 [36.0–54.0]	
High school	54	71.5 [62.0–81.5]		47.5 [35.2–51.0]	
Technical/vocational	13	74.0 [69.0–81.0]		48.0 [41.0–53.0]	
Vocational	14	75.5 [71.2–79.2]		48.0 [44.8–48.0]	
Graduate	1	75.0 [75.0–75.0]		40.0 [40.0–40.0]	
**Religion**			0.501		0.188
Catholic	131	73.0 [63.5–81.5]		47.0 [36.0–51.0]	
Jehovah’s witness	16	68.5 [61.5–78.2]		51.0 [38.5–73.5]	
Evangelical	36	72.0 [63.8–78.0]		47.5 [34.8–55.0]	
**Marital status**			<0.001		0.016
Single	23	64.0 [58.0–74.5]		51.0 [43.5–57.0]	
Married	67	74.0 [69.5–80.0]		48.0 [41.0–53.0]	
Cohabiting	75	74.0 [63.0–86.0]		42.0 [32.0–51.0]	
Separated	9	58.0 [55.0–66.0]		42.0 [38.0–80.0]	
Widowed	9	70.0 [63.0–93.0]		44.0 [22.0–50.0]	
**Healthcare system**			0.028		0.797
Subsidized	141	73.0 [65.0–80.0]		47.0 [36.0–53.0]	
Contributory	23	72.0 [63.0–87.0]		47.0 [36.5–50.0]	
Special	19	62.0 [61.0–71.5]		48,0 [39.0–53.0]	
**Socioeconomic status**			0.383		0.088
Stratum 1	122	72.0 [64.0–78.0]		48.0 [39.2–53.0]	
Stratum 2	50	72.0 [62.0–84.5]		41.0 [32.8–50.8]	
Stratum 3	11	77.0 [69.0–85.5]		42.0 [35.0–52.0]	
**Occupation**			0.064		0.233
Housewife	24	78.5 [70.0–88.8]		44.0 [33.2–53.2]	
Student	3	62.0 [50.0–68.5]		56.0 [51.5–64.0]	
Employed	48	73.5 [67.0–79.2]		46.5 [35.0–51.0]	
Unemployed	108	70.0 [62.0–78.0]		47.5 [38.0–54.0]	
**Underlying conditions**			0.091		0.049
AH + CKD	89	72.0 [64.0–82.0]		47.0 [38.0–51.0]	
DM2 + AH + CKD	60	70.5 [62.0–78.0]		48.0 [37.5–54.2]	
DM2 + CKD	21	73.0 [67.0–76.0]		47.0 [34.0–49.0]	
AH + DM2 + CKD + heart disease	3	67.0 [61.0–72.0]		86.0 [64.0–104.5]	
AH + CKD+ thyroid disease	3	75.0 [66.5–84.5]		40.0 [36.0–40.5]	
AH + CKD + lupus	5	93.0 [88.0–95.0]		32.0 [30.0–37.0]	
AH + CKD + arthritis	2	80.5 [73.8–87.2]		52.5 [51.8–53.2]	
**Complications related to the hemodialysis process**			0.001		<0.001
None	100	76.5 [67.0–86.2]		43.0 [33.8–51.2]	
Infection	57	71.0 [62.0–77.0]		49.0 [45.0–55.0]	
Blood clots	14	62.5 [60.5–71.8]		39.5 [20.5–46.8]	
Bleeding	12	70.0 [61.0–72.0]		48.0 [43.5–54.5]	

Note: Values are expressed as medians [interquartile range]. Comparisons between two groups were performed using the Mann–Whitney U test, and comparisons between three or more groups using the Kruskal–Wallis test. *p*-values are presented in exact format.

**Table 5:** Analysis of the correlation between sociodemographic and clinical variables and MOS-SSS and GCPC-UN scores (*n* = 183).

Variable	*n*	MOS-SSS Total (ρ)	*p*-Value	GCPC-UN Total Frequency (ρ)	*p*-Value
Age	183	−0.005	0.946	−0.148	0.046
Duration of hemodialysis (months)	183	0.087	0.240	0.030	0.686

Note: ρ—Spearman’s correlation coefficient. The *p*-values are presented in exact format.

**Table 6:** Multivariate model for the prevalence of chronic disease burden (total GCPC-UN score) (*n* = 183).

Variable	β-Adjusted	95% CI	*p*-Value
Total MOS-SSS (for every 10 points)	−3.216	−7.054 to 0.622	0.101
Age (for every 10 years)	−2.09	−4.322 to 0.142	0.067
Male gender	2.094	−2.836 to 7.024	0.405
Rural origin	−3.781	−9.844 to 2.282	0.222
Single	4.339	−4.607 to 13.285	0.342
Low socioeconomic status (strata 1)	3.938	−1.014 to 8.891	0.119
Duration of hemodialysis (for every 12 months)	0.628	0.022 to 1.234	0.042
DM2	1.881	−3.280 to 7.042	0.475
Other comorbidities	−3.958	−11.782 to 3.867	0.322
Any complications related to hemodialysis	2.68	−1.773 to 7.134	0.238

Note: Multivariate linear regression model with robust errors (HC3). Adjusted β: adjusted regression coefficient; 95% CI: 95% confidence interval. *p* -values are presented in exact format. Type 2 diabetes (T2D) complications (T2D + HTN + CKD, T2D + CKD, and HTN + T2D + CKD + heart disease); and (3) other comorbidities, which included HTN + CKD + thyroid disease, HTN + CKD + lupus, and HTN + CKD + arthritis. Marital status with a partner (married/cohabiting) and without a partner (single/separated/widowed). Complications were recorded as no complications vs. some complications.

## Data Availability

Dataset available on request from the authors.

## Public Involvement Statement

There was no public involvement in any aspect of this research.

## Guidelines and Standards Statement

This manuscript was drafted in accordance with the Strengthening the Reporting of Observational Studies in Epidemiology (STROBE) guidelines for cross-sectional studies.

## Abbreviations

The following abbreviations are used in this manuscript:

DM2	Type 2 diabetes mellitus
SD	Standard deviation
CKD	Chronic kidney disease
GCPC-UN	The Burden of Chronic Illness on the Patient
AH	Arterial hypertension
CI	Confidence interval
MOS-SSS	Medical Outcomes Study Social Support Survey
IQR	Interquartile range
STROBE	Strengthening the Reporting of Observational Studies in Epidemiology
